# The association between occupational stress and psychosomatic wellbeing among Chinese nurses: A cross-sectional survey

**DOI:** 10.1097/MD.0000000000015836

**Published:** 2019-05-31

**Authors:** Bo Gu, Qiling Tan, Shangping Zhao

**Affiliations:** The Third Comprehensive Care Unit, West China Hospital, Sichuan University, Chengdu, Sichuan, P.R. China.

**Keywords:** anxiety, depression, nurse, occupational stress, sleep quality, somatic symptoms

## Abstract

Occupational stress impairs nurses’ psychosomatic wellbeing, which includes anxiety, depression, sleep quality, and somatic symptoms; however, few studies have focused on the associations between the subdimensions of occupational stress [workload and time pressure (WTP), professional and career issues (PC), patient care and interaction (PCI), interpersonal relationships and management problems (IRMP), resource and environment problem (REP)] and psychosomatic wellbeing among nurses in China. This study thus examined these associations using a cross-sectional survey in Sichuan, China. An online application was devised to collect data, with the scales of sociodemographic and occupational variables, Nurse Job Stressor Questionnaire, the 9- and 15-item Patient Health Questionnaires, the 7-item Generalized Anxiety Disorder scale, and the Pittsburgh Sleep Quality Index. Investigation was completed by 2889 nurses (96.7% women; mean age = 31.20 ± 6.72 years). Relationships were identified by correlation and multivariate regression analyses. Most (68.3%) nurses had high levels of occupational stress. The multivariate analyses revealed that WTP was correlated with anxiety (*P* = .003). PC was associated with depression (*P* = .033) and sleep quality (*P* = .078). PCI was correlated with anxiety (*P* = .031) and somatic symptoms (*P* = .005). IRMP was associated with anxiety (*P* = .018), depression (*P* = .001), and somatic symptoms (*P* = .025). Lastly, REPs had nonsignificant relationships with depression, anxiety, sleep quality, and somatic symptoms. In sum, nurses had high levels of occupational stress; therefore, a series of strategies should be implemented to help nurses cope with the above issues, which could promote nurses’ psychosomatic wellbeing, and have a buffering effect on nurses’ depression, anxiety, poor sleep quality, and somatic symptoms.

## 1 Introduction

Nurses face various work-related stressors. Empirical studies have shown that occupational stress not only impairs nurses’ psychosomatic wellbeing, but it is also a risk factor for patient safety and nursing quality.^[1,2]^ Following the nursing field has been innovated in the last 2 decades, nurses have taken more responsibilities on caring patients and communities than before. As a result, nurses work in a competitive and complex environment. The situation of work-related stress was easily underestimated and coping to stress was used weakly in developing countries.^[3]^ In China, the contradictions between nursing care and patients’ expectation have increased, and the relationship between nurses and patients has become increasingly intense, resulting in a variety of potentially stressful or hazardous circumstances for nurses.^[4]^ Nurses are vulnerable to occupational stress, which has direct and indirect negative consequences for nurses’ health and patient outcomes.^[5]^

Previous studies indicated that nurses with increased occupational stress had a higher risk of unhealthy psychosomatic wellbeing (e.g., anxiety, depression, distress, lower back pain, headache, and appetite loss),^[6–8]^ poor sleep quality,^[9]^ and cardiovascular diseases^[10]^ compared to those with less occupational stress. These stress-related psychosomatic problems are associated with an increased risk of poor health and disease. The allostatic load model of stress posits that persistently elevated stress will result in psychological and physical health problems if the individual lacks the skills to appropriately adapt.^[10]^ In this model, depression, anxiety, somatic symptoms, and sleep quality are all associated with stress, and increased stress is associated with an increased risk of cardiovascular diseases and mortality.^[11]^ However, this model have not shown the relationship between subdimensions of stress and psychosomatic wellbeing.

Considering that the prevalence of stress-related diseases is increasing,^[12]^ close attention should be paid to occupational stress. Several researchers have focused on occupational stress hazards (e.g., job control,^[13]^ social support,^[13]^ family-work balance,^[13]^ workload,^[14]^ and coping styles^[15]^); however, few studies focused on the associations between the subdimensions of occupational stress and psychosomatic wellbeing among nurses in China. In sum, previous studies did not present a convincing picture of the correlations between the subdimension of occupational stress and nurses’ psychosomatic symptoms. While the allostatic load model provides useful insight into the relationship between occupational stress and psychosomatic symptoms, our hypotheses are that each subdimension of occupational stress among nurses will be distinctly associated with parts of psychosomatic symptoms.

## 2 Methods

### 2.1 Population and study design

A cross-sectional survey was used. Inclusion criteria were registered nurses who were working in public comprehensive hospitals and voluntary to participate. Nurses who were pregnant, breastfeeding, or on sick leave were excluded. Nurses were enrolled using stratified cluster sampling from 7 hospitals (1% of the public comprehensive hospitals) across 5 regions [east (n = 1), south (n = 1), west (n = 1), north (n = 1), and central (n = 3)] in Sichuan, China.

Participants’ anonymity was ensured; questionnaires were completed voluntarily; and online, informed consent was obtained before survey commencement. Ethical approval was granted by the Ethics Committee of West China Hospital, Sichuan University.

### 2.2 Data collection and instruments

An online application supported by WeChat, which was created by an Internet company in China, was used to collect survey data. The application was open from September to October 2017 in Sichuan Province, P.R. China. Varied instruments were included in the application including the Nurse Job Stressor Questionnaire (NJSQ), the 9- and 15-item Patient Health Questionnaires (PHQ-9 and PHQ-15, respectively) from the *Diagnostic and Statistical Manual of Mental Disorders, Fifth Edition (DSM-5)*, the 7-item Generalized Anxiety Disorder (GAD-7) scale, and the 19-item Pittsburgh Sleep Quality Index.

To ensure an adequate sample size and valid response rate, nursing directors of sampled hospitals agreed to implement this survey in their hospitals during the recruitment process. Meanwhile, to ensure collecting reliable data, participants had known they would receive a personal report that show all scales’ status and coping method ahead of enrolling in this study, which was sent to their personal mobile phones as soon as they finished the investigation.

#### 2.2.1 Sociodemographic and occupational variables

Sociodemographic variables include sex, age, marital status, living status, and educational degree. Occupational variables include work experience, hospital level (Hospital level in China is evaluated by the number of beds, scientific research capacity, staffing, medical and nursing quality, and so on. There are A-B-C and 3-2-1 rankings, and 3A and 2A are top 2 of all hospital levels), professional title (nurse, senior nurse, charge nurse, associate, or chief nurse), frequency of night shift per month, and coping with pressure (easy to cope, stressful to cope).

#### 2.2.2 Occupational stress

Occupational stress was assessed with the NJSQ. The NJSQ is a 35-item questionnaire, which was developed by Li and Liu in 2000.^[16]^ It is widely used in China and has been shown to be both reliable and valid.^[16]^ The scale consists of 5 subscales: professional and career issues (PC; 7 items; e.g., “you had little opportunity to further study”), workload and time pressure (WTP; 5 items; e.g., “your workload is too heavy”), resource and environment problem (REP; 3 items; e.g., “you had a bad working environment”), patient care and interaction (PCI; 11 items; e.g., “you are always worried about the potential for nursing errors and adverse events”), and interpersonal relationships and management problems (IRMP; 9 items; e.g., “there was poor cooperation during your work”).

In this study, Cronbach alphas were 0.91 for PC, 0.81 for WTP, 0.92 for REP, 0.94 for PCI, 0.90 for IRMP, and 0.93 for the entire scale. Participants responded using a 5-point Likert scale (1 = *strongly disagree* to 5 = *strongly agree*). Mean scores were calculated with 2.5 as a cut-off value: ≥2.5 indicated high stress and <2.5 indicated low stress (i.e., higher scores indicated higher occupational stress).

#### 2.2.3 Psychosomatic wellbeing

We assessed psychosomatic wellbeing (depression, anxiety, somatic symptoms, and sleep quality) with varied measures. Depression was assessed with the PHQ-9. Anxiety was assessed with the GAD-7. The participants responded to both questionnaire using a 4-point scale (0 = *never*, 2 = *<6 days*, 3 = *>7 days*, and 4 = *almost every day*). Total scores on the PHQ-9 range from 0 to 27, which indicate no depression (0–4), mild depression (5–9), moderate depression (10–14), moderate to severe depression (15–19), or severe depression (20–27). Total scores on the GAD-7 range from 0 to 21, which indicate no anxiety (0–5), mild anxiety (6–10), moderate anxiety (11–15), and severe anxiety (16–21). Both instruments are reliable and valid.^[17,18]^

Somatic symptoms in the last month were assessed with the PHQ-15 from the *DSM-5*. Responses were provided using a 3-point scale (0 = *never*, 2 = *a few days*, and 3 = *always*). Total scores ranged from 0 to 30, which indicate no somatic symptoms (0–4), mild somatic symptoms (5–9), moderate somatic symptoms (10–14), and severe somatic symptoms (≥15). This scale is valid and reliable for health screening.^[19]^

Sleep quality was assessed with the 19-item Pittsburgh Sleep Quality Index. The Cronbach internal consistency reliability was 0.845, and the retest reliability of the whole scale was 0.994.^[20]^ Higher scores indicate worse sleep quality. Sleep quality was quantified as follows: perfect sleep quality (0–5), good sleep quality (6–10), poor sleep quality (11–15), and severely poor sleep quality (16–21).

### 2.3 Statistical analyses

All analyses were performed with SPSS 16.0 (SPSS Inc, Chicago, IL). Significance was defined as *P* < .05. For descriptive analyses, mean and standard deviation or median and interquartile range were used for continuous variables, and number and percentage were used for categorical variables. Pearson correlations, chi-square tests, and the ordinary least squares regression as linear regression was carried out to assess the relationship between subdimensions of occupational stress and depression, anxiety, physical symptoms, and sleep quality.

## 3 Results

### 3.1 Nurses’ sociodemographic characteristics

Of the 3500 nurses who were recruited, valid questionnaires were obtained from 2889 nurses (valid response rate = 82.54%). Enrolled nurses had a mean age of 31.20 ± 6.72 years and had a median work experience of 7 years (interquartile range = 4–12 years). Most nurses were women (96.7%), married (67.5%), living with family (75.3%), and had at least a bachelor's degree (53.8%). Most (67.6%) nurses found it difficult to cope with occupational stress. Participants’ characteristics are shown in Table 1.

**Table 1:**
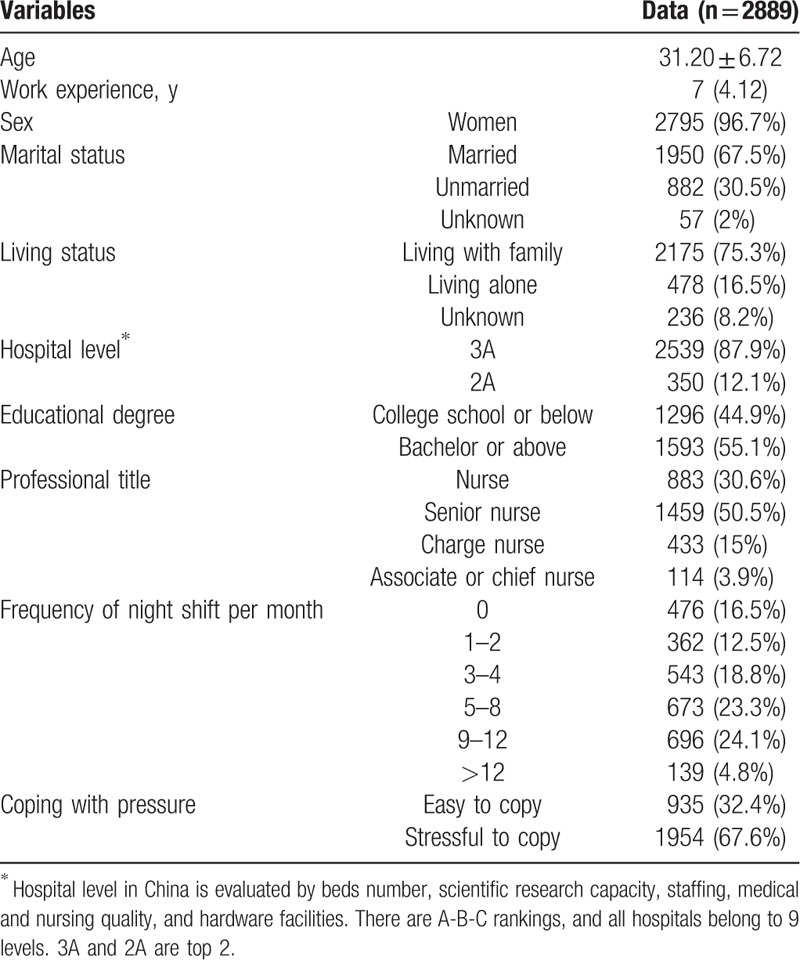
Sociodemographic and occupational variables of nurses.

### 3.2 Nurses’ occupational stress and psychosomatic wellbeing

Nurses’ mean occupational stress score was 2.77 ± 0.60, and 68.3% of nurse had high occupational stress. Scores from highest to lowest per subdimension were as follows: WTP (3.26 ± 0.87), REP (2.97 ± 1.01), PC (2.96 ± 0.72), PCI (2.79 ± 0.64), and IRMP (2.26 ± 0.74). Both median anxiety and depression scores were 5. Mean somatic symptom and sleep quality scores were 7.22 ± 5.34 and 7.61 ± 3.50, respectively. Results thus revealed that nurses on average had no anxiety, mild depression, mild somatic symptoms, and good sleep quality. Nurses who were classified as having high occupational stress had worse anxiety, depression, somatic symptoms, and sleep quality than did nurses with low occupational stress (Table 2). In addition, the *R*^2^ for models on depression, anxiety, sleep quality, and somatic symptom were 0.647, 0.585, 0.341, and 0.385, respectively.

**Table 2:**
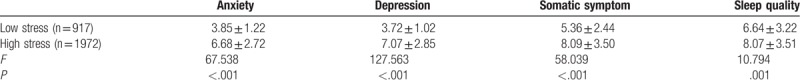
The difference of psychosomatic wellbeing in high and low stress.

### 3.3 Relationship between the subdimensions of occupational stress and psychosomatic wellbeing

Pearson correlation analyses showed that the subscales of occupational stress were positively associated with depression, anxiety, somatic symptoms, and sleep quality (Table 3). Results indicated that occupational stress and psychosomatic wellbeing had an interactional relationship. Table 4 also shows the results of the ordinary least squares regression analysis (notably, IRMP-predicted depression, anxiety, and somatic symptoms; PC-predicted depression; WTP-predicted anxiety; and PCI-predicted anxiety and somatic symptoms). REP had no significant relationships with depression, anxiety, sleep quality, or somatic symptoms. Sex, marital status, work experience, and frequency working the night shift per month predicted parts of psychosomatic wellbeing; however, age, education, and professional title had no significant relationship with psychosomatic wellbeing.

**Table 3:**
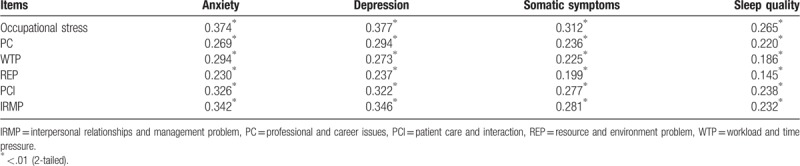
Pearson correlation between occupational and psychosomatic wellbeing.

**Table 4:**
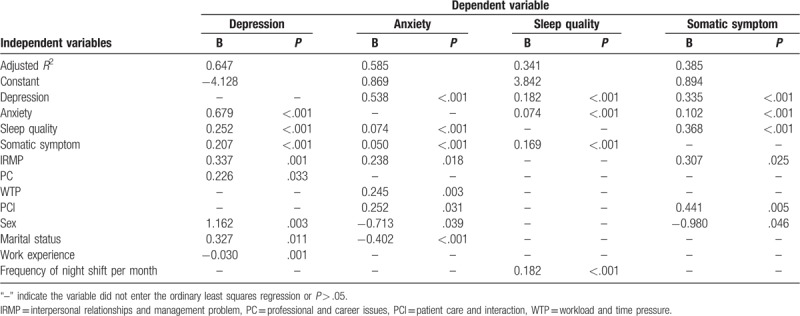
The ordinary least squares regression of psychosomatic wellbeing.

## 4 Discussion

### 4.1 Nurses’ occupational stress

Occupational stress challenges workers’ ability to cope with unmatched work demands, and personal knowledge.^[21]^ Our study showed that nurses in China had high levels of occupational stress. Among the subscales of the NJSQ, the score of WTP was the highest and IRMP was the lowest. These results are inconsistent with Li and Liu's findings in 2000.^[16]^ Simpson et al^[22]^ also found that nurses had high levels of occupational stress because of inadequate nursing staffing. In China, a standard 1:4 nurse-patient ratio was reached; however, they included nurses on sick leave and nurse interns. In fact, each nurse from tertiary hospitals is typically responsible for 8 or more patients. Furthermore, Chinese medical reform has implemented ways to deter patients from visiting 3A-level hospitals (i.e., hospitals considered to be the best in China) and instead seek care at first clinical hospitals (i.e., town- or county-level public hospitals) to use medical resources effectively. Moreover, nurses spend extra energy and time educating themselves, conducting research, and tutoring interns, which also may increase their occupational stress.

Most nurses in the current study were young to middle-aged, women, married, and living with family. As ^Nohe^ et al's^[23]^^meta-analys^i^s showed, work-family conflict is^ reciprocally correlated with occupational stress, and women typically devote a significant amount of time to family in Chinese traditional culture.^[24]^ Therefore, the work-family conflict may have made nurses feel stressed. In contrast, PC scores in the current study were better than those previously reported,^[7]^ possibly because nurse in China are more specialized and acquire more professional development; therefore, these ambitious nurses display decreased occupational stress.

Empirical evidence has also shown how problematic it is to decrease healthcare costs and reduce the number of nurses.^[25,26]^ Therefore, adequate nurse staffing is fundamental to decrease nurses’ work load and occupational stress, which will likely lead to better patient outcomes.^[27,28]^ Moreover, nursing managers should provide flexible shift arrangements when work-family conflict occurs.^[29,30]^ Furthermore, lifelong learning and balancing work-family conflicts can be used to cope with occupational stress.

### 4.2 Relationship between dimensions of occupational stress and psychosomatic wellbeing

As posited in the allostatic load model, high stress levels were associated with increased anxiety, depression, somatic symptoms, and poor sleep quality. However, more specifically, only some dimensions of occupational stress were associated with psychosomatic wellbeing. According to the results of regression models, depression had a series of predictors which were men, anxiety, poor sleep quality, somatic symptom, IRMP, PC, not married, and shorter of work experience. Then women, married, depression, poor sleep quality, somatic symptom, IRMP, WTP, and PCI were predictors of anxiety. Moreover, the more serious of depression, anxiety, somatic symptom, and more night shift indicated poorer sleep quality, and women, depression, anxiety, poor sleep quality, IRMP, and PCI were predictors of somatic symptom.

REP showed no significant relationships with depression, anxiety, sleep quality, or somatic symptoms. In China, nursing equipment and instrument are always equipped and updated well in 3A and 2A hospitals. Therefore, there is no need for nurses to suffer stress from REP. On the contrary, dimensions of occupational stress had no significant relationship with sleep quality. Sleep quality would be improving by less frequency of night shift per month.

The highest stressor in our study—WTP—only could be a predictor for anxiety. Increasing workload of nurses makes it hard to provide high-quality nursing services and keeps patient safety. As a result, it cannot satisfy patients’ increasing needs. Anxiety is a subjective feeling of uneasiness and worry.^[31]^ Therefore, the risk of nursing adverse events and potential of patients’ complaints is accelerating nurses’ anxiety. However, Dalri et al^[32]^ found that workload had no relationship with psychological stress. Therefore, to reduce anxiety, balancing the workload should be attentively considered and coping to stress should be learnt.

Increased PC was associated with increased depression. According to social stereotypes, it is easy to understand why nurses would have depression, especially when disrespected by patients and patients’ families. Moreover, nurses have a desire to pursue further professional development because it concerns their compensation package. However, most had little time to do research or further their education during work time. Because most nurses were married, work-family conflict likely also caused a lot of stress and adversely affected participants’ mood. Therefore, a clear career development and progression plan to nurses should be implemented.

PCI was correlated with anxiety and somatic symptoms, which was consistent with prior results.^[16]^ PCI comprises 2 core contents, which can exacerbate nurses’ somatic symptoms: first, exaggerated medical stories can lead to negative perceptions of nurses and second, the potential of adverse events can promote anxiety. Therefore, effective prevention and management of nursing adverse events should be implemented, and a supportive organizational environment and patient safety culture should be promoted.

IRMP was associated with anxiety, depression, and somatic symptoms. IRMP concerns interpersonal relationships between nurses and their colleagues. Although IRMP was the least associated with occupational stress among all the subdimensions, it was still related with all psychosomatic wellbeing indicators except sleep quality. A prior study indicated that interpersonal relationships and perceived social support had direct effects on psychological symptoms such as depression.^[33]^ Conversely, good interpersonal relationships were indicative of a healthy and satisfying environment,^[34]^ which is advantageous for nurses’ health. Therefore, nurse managers should cultivate nurses’ interpersonal communication skills and create a healthy work environment.

## 5 Limitation

This study was a cross-sectional survey, which showed a time-point status without causal relationship. If nurses happen to have some problems during work, occupational stress could be varying. Thus, a cohort research should be done. Moreover, all nurses participated in this study voluntarily. As a matter of fact, we could not avoid sample bias if someone with high occupational stress refused to participate, even we did research quality control during all survey period.

## 6 Conclusions

Nurses had high levels of occupational stress; specifically, WTP, PC, PCI, and IRMP were correlated with distinct parts of psychosomatic wellbeing; however, because we employed a cross-sectional design, causal relationships cannot be inferred. A series of strategies that promote nurses’ coping abilities are needed to increase nurses’ psychosomatic wellbeing. Increasing the number of nurses might also be an effective way to foster high-quality nursing in China. Furthermore, strengthening nurses’ interpersonal communication skills may help offset occupational stress, which might buffer the effects of depression, anxiety, and poor sleep quality. Lastly, nurses’ psychosomatic health should be considered by administrative leaders, because it is closely related to patients’ outcomes.

## Acknowledgments

The authors thank all members of the Nursing Association of Sichuan Province and Beijing Haola Technology Co, Ltd for their technological support with the mobile network survey and for providing immediate individual assessment reports. The authors also thank Professor Song for statistical analysis consulting and appreciate all head nurses who helped with data collection. Lastly, they give their heartfelt thanks to all participating nurses. The authors also thank for English-language editing by Dr Zhang, Yuxi Zheng, and Editage sincerely.

## Author contributions

**Conceptualization:** Qiling Tan.

**Data curation:** Shangping Zhao.

**Investigation:** Qiling Tan.

**Methodology:** Bo Gu.

**Project administration:** Bo Gu, Shangping Zhao.

**Supervision:** Bo Gu.

**Writing – original draft:** Bo Gu.

**Writing – review and editing:** Qiling Tan, Shangping Zhao.

Shangping Zhao orcid: 0000-0002-5780-1437.
